# No relationship between autistic traits and salivary testosterone concentrations in men from the general population

**DOI:** 10.1371/journal.pone.0198779

**Published:** 2018-06-14

**Authors:** Diana Weiting Tan, Murray T. Maybery, Michael W. Clarke, Renata Di Lorenzo, Melissa O. Evans, Michael Mancinone, Christina Panos, Andrew J. O. Whitehouse

**Affiliations:** 1 Neurocognitive Developmental Unit, School of Psychological Science, University of Western Australia, Perth, Western Australia, Australia; 2 Telethon Kids Institute, University of Western Australia, Perth, Western Australia, Australia; 3 Biological and Molecular Mass Spectrometry Facility, Centre for Microscopy, Characterisation and Analysis, University of Western Australia, Perth, Western Australia, Australia; Univdersity Hospital of TübingenUniversitatsklinikum Tubingen, GERMANY

## Abstract

It is suggested that testosterone may play a part in the higher prevalence of Autism Spectrum Disorder (ASD) in males compared to females. Previous studies have reported elevated postnatal testosterone levels in children and women with ASD but not in men. We compared levels of salivary testosterone across 67 undergraduate males (*M*_age_ 19.5 yrs, *SD* 1.92) selected for low, mid-range and high levels of autistic traits assessed using the Autism-spectrum Quotient. Analyses revealed no significant differences in testosterone concentrations across the three groups. The current data add to the increasing evidence for the lack of relationship between autistic traits and postnatal levels of testosterone in men.

## Introduction

Testosterone is a biologically potent sex steroid that has masculinising effects on the brain and behaviour [1]. The two- to four-fold increase in the prevalence of Autism Spectrum Disorder (ASD) in males relative to females [2,3] is suggested to be related to heightened testosterone exposure during prenatal life [4]. However, evidence for this relationship is inconclusive. While elevated levels of prenatal testosterone have been found to be linked to more pronounced autistic traits in typically-developing children [5,6], not all studies have identified this link in other neurotypical populations [7,8]. A recent study of autistic and non-autistic boys reported that the two groups had similar levels of prenatal testosterone but the ASD group showed increased steroidogenic activity when the levels of cortisol and steroids involved in the biosynthesis of testosterone were analysed [9].

Whilst early exposure to testosterone is more likely to have enduring effects in neurodevelopment [10], broader neurocognitive research is beginning to acknowledge that postnatal testosterone has lasting albeit impermanent effects on behaviours [11]. Postnatal testosterone levels are commonly derived from blood serum or saliva samples. Blood serum is considered to be the ‘gold standard’ for steroid analyses because it is thought to reflect more biologically active compounds than saliva [12]. Nevertheless, validation studies have consistently found salivary testosterone to be highly correlated with serum testosterone [13,14].

Several studies have investigated the relationship between postnatal testosterone concentrations and ASD. Majewska et al. [15] reported that salivary testosterone levels of prepubescent boys and girls with ASD were significantly elevated compared to those of age-matched controls. Other studies reported 2.23 times increase in salivary testosterone [16] and 2.1 times increase in serum testosterone levels [17] among autistic boys compared to typically-developing boys.

To our knowledge, only three studies have investigated postnatal steroid profiles of adults diagnosed with ASD. Compared to their same-sex counterparts, two of the three studies reported heightened serum testosterone levels among autistic women [18,19] while one study found increased androstenedione but not testosterone levels in autistic men and women [20]. None of the three studies observed a relationship between testosterone and autistic traits in men.

The investigation of autistic traits in the general population is a methodological approach gaining traction in autism research [21]. There is accumulating evidence that autistic traits are on a continuum in the wider community, with ASD representing an extreme end of the distribution [22]. Understanding developmental factors that are associated with population variation in autistic traits may shed light into the biological mechanisms underlying clinical ASD. Takagishi et al. [23] examined salivary testosterone levels among neurotypical men and women (21–68 year-old) in relation to autistic traits measured by the Autism-spectrum Quotient (AQ) [24]. Testosterone levels were unrelated to AQ scores in men and in women after statistically controlling for age. Because hormone levels are known to vary with age [25], salivary testosterone obtained from individuals across lifespan in Takagishi et al. [23] limits our ability to interpret these findings.

The current study investigated salivary testosterone concentrations and autistic traits in a population of undergraduate students in Australia. As a large proportion of young female adults in Australia use a contraceptive pill [26] which is known to affect baseline testosterone concentrations [25], only males were included in this study. The present study advances existing knowledge in two ways. First, we sampled a young adult population within a constrained age band of 17–25 yrs, which will reduce the likelihood of age-related changes in testosterone levels. Second, Takagishi et al. [23] adopted a continuous AQ design to examine the correlation between AQ scores and salivary testosterone concentrations. However, a recent meta-analysis found that continuous AQ designs have less statistical power compared to quantile AQ designs [27]. Therefore, we employed the quantile design which involved selecting groups of individuals with varying levels of autistic traits based on the score distribution of AQ administered to a large number of people.

## Material and methods

### Participants

A total of 1,995 undergraduate students completed the AQ. Based on the lower, middle and upper 15% of the AQ score distribution, students with low (AQ ≤ 12), mid-range (15 ≤ AQ ≤ 18) and high (AQ ≥ 23) scores were invited to participate further. Twenty-five men in each AQ group (M_age_ = 19.6 yrs, *SD* = 1.99) took part in the study and were identified as post-pubertal, as measured by the Puberty Developmental Scale [28]. All participants provided informed written consent.

### Autism-spectrum Quotient [24]

The AQ is a self-report questionnaire that measures levels of autistic traits in the general population and has good test-retest reliability and validity [29]. This instrument consists of 10 statements for each of its five subscales—‘Attention to Details’, ‘Attention Switching’, ‘Communication’, ‘Imagination’, Social Skills’. Participants indicated whether they strongly agree, agree, disagree or strongly disagree with each statement and the AQ score ranges from 0 to 50.

### Saliva samples procurement and analysis

Participants provided their body mass index and indicated whether, in the previous 12hr, they had consumed a) alcohol, b) caffeine, c) nicotine, or d) medications, or carried out vigorous activities. Participants also reported any endocrinological disorders such as congenital adrenal hyperplasia and/or existing oral health issues. To control for diurnal variations, saliva samples were collected in the morning. Participants fasted for an hour and rinsed their mouths 15min before saliva sampling. Using passive drool method, participants provided 10mL of saliva which were kept frozen at –20°c until analysis.

During analysis, saliva samples were thawed and prepared at room temperature before being injected into the liquid chromatography-tandem mass spectrometry (LC-MS/MS). Free testosterone concentration was measured in pmol/L. Further details related to this assay method are described in the Supporting Information file (S1 File).

### Statistical analyses

A single-factor between-subject ANOVA supplemented by *t*-tests were used to test whether there was a significant effect of AQ group on salivary testosterone levels. Pearson correlation analyses were employed to investigate the relationships between testosterone concentrations, total AQ scores and the five subscales of AQ.

### Ethics statement

The recruitment and testing of all participants were conducted in accordance with the ethical approval obtained for this study from the Human Research Ethics Committee at the University of Western Australia (reference number: RA/4/1/6668). Informed written consent was obtained from all individual participants included in the study.

## Results

Eight participants reported oral health issues and were excluded from the analysis due to possible blood contamination in the saliva samples. A final sample of 67 participants were included in the analyses. Chi-square analyses revealed no significant differences in the factors that could interfere with salivary hormone analysis (e.g., caffeine intake) across the three AQ groups (all *p*s >.05; frequency of factors is provided in the Supporting Information file, S1 Table). Table 1 below provides participants’ demographic information.

**Table 1:** Descriptive statistics of participants’ demographic information, AQ scores and salivary testosterone concentrations.

Variables	Statistics	Low-AQ	Mid-AQ	High-AQ
Age (yrs)	*M*	19.27	19.48	19.82
	*SD*	1.86	2.13	1.79
	Range	18–25	18–25	17–24
BMI	*M*	22.34	22.82	22.89
	*SD*	2.36	3.08	2.72
	Range	18.7–27.6	17.7–28.3	16.9–28.9
AQ	*M*	9.05	16.49	25.64
	*SD*	1.84	1.12	3.43
	Range	5–12	15–18	22–35
Testosterone (pmol/L)	*M*	319.1	350.3	318.3
	*SD*	67.2	102.7	88.9

**Fig 1:**
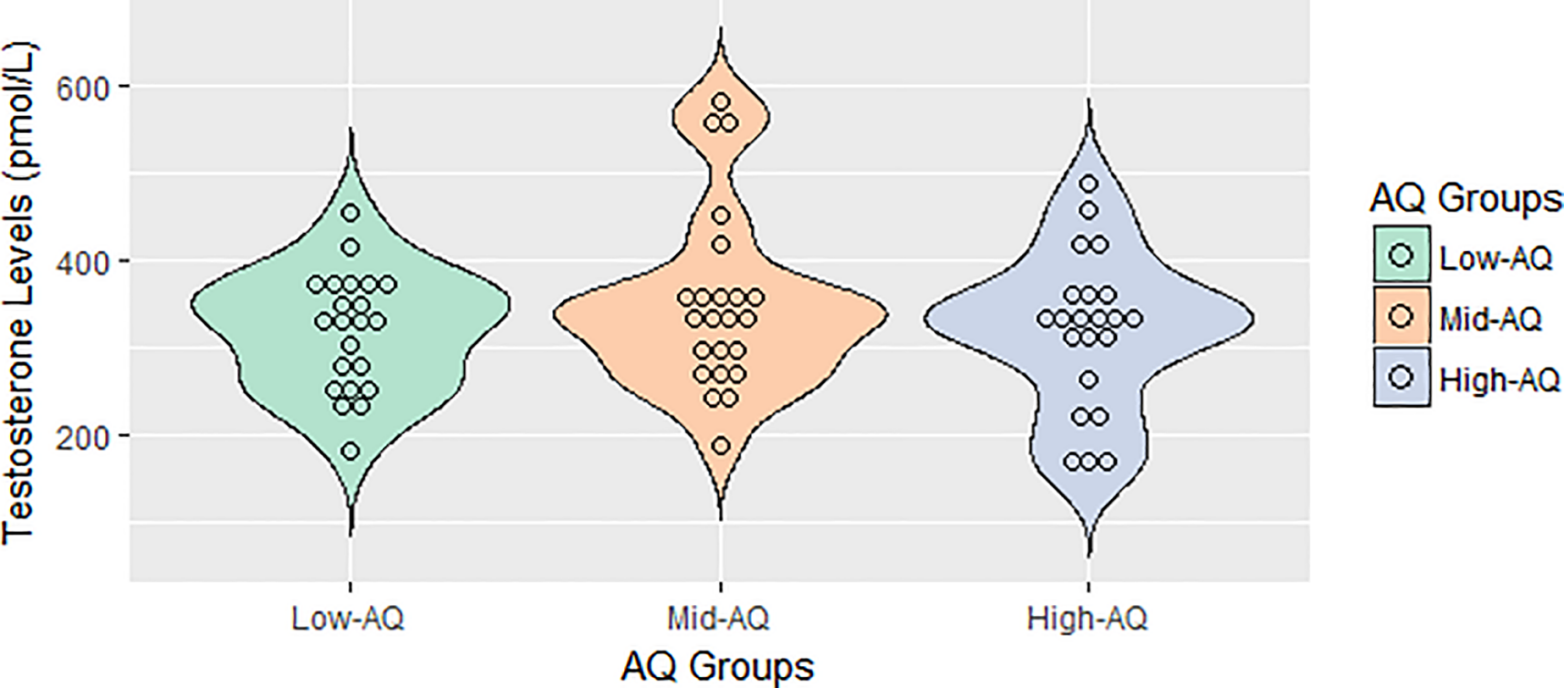
Testosterone level of each participant is presented as an unfilled circle. The outlines of the violin plot illustrate the kernel probability density.

Shapiro-Wilk normality test and Levene’s test for homogeneity of variance indicated that the assumptions of parametric tests were not violated for the testosterone measure (both *p*s > .05). ANOVA showed that salivary testosterone concentrations were not significantly different across the three AQ groups, *F*(2,64) = 0.78, *p* = .46 (Fig 1). The difference in testosterone concentrations between the two extreme groups (low- *vs*. high-AQ) was also not statistically significant, *t*(42) = 0.24, *p* = .81.

Pearson correlational analyses revealed no statistically significant relationships between testosterone concentrations, total AQ score, and the five subscales of AQ (all *p*s>.05; Table 2).

**Table 2:** Correlations between log-transformed salivary testosterone concentrations, total AQ scores, and the five subscales of AQ.

	Total	Subscales				
	AQ	Attention to Details	Attention Switching	Communication	Imagination	Social Skills
Testosterone	.04	.08	.001	–.02	–.006	.06

## Discussion

The current study built on Takagishi et al. [23] through the use of a constrained age band and a statistically robust quantile-AQ design. Testosterone concentrations did not differ between men with low, mid-range and high levels of autistic traits. Consistent with Takagishi et al., there was no statistically significant correlation of salivary testosterone concentrations with total AQ scores or with any of the AQ subscales.

The present findings suggest two implications on the relationship between testosterone concentrations and the extent of autistic traits in men. First, an early review presented behavioural and cognitive findings suggesting that prenatal exposure to androgen may be linked to an exaggeration of ‘male-typical’ traits in ASD [30]. Several endocrinological studies has provided some evidence for an increase in the biosynthesis of androgen during very early foetal life among boys who were later diagnosed with ASD [9], as well as elevated levels of postnatal salivary and serum testosterone in prepubescent boys with ASD [15–17].

However, past studies have consistently found no difference in the testosterone concentrations between post-pubertal men with and without ASD [18–20], and between neurotypical men with high and low levels of autistic traits [23] (including present study). Evidence obtained during prenatal and postnatal (pre- and post-puberty) life suggests an age-dependent variation in the association between testosterone and autistic traits. It is possible that puberty, a transitional period of development from childhood to adulthood, may be involved in normalising elevated levels of prepubertal testosterone associated with autistic traits.

The second implication is that the link between postnatal testosterone and autistic traits in adulthood may also be sex-specific given that increased testosterone levels are more frequently reported in women with ASD compared to men [18,19] though higher androstenedione levels have been reported for both men and women with ASD relative to neurotypical controls [20]. This accords with several lines of evidence associated with androgen-driven characteristics in women with clinical or subclinical levels of autistic traits. Androgen-driven characteristics include increased prevalence of steroidopathic conditions such as hirsutism [31] and less feminine facial morphology [32,33]. Further investigation into testosterone concentrations of groups of women selected for varying levels of autistic traits who are not contraceptive pill users is warranted.

### Conclusion

Despite the use of a narrow age bracket and a quantile-AQ design, the current investigation did not observe an association between autistic traits in neurotypical men and salivary testosterone concentrations. The present findings contribute to the growing evidence for no association between autistic traits and post-pubertal levels of testosterone in men.

## Supporting information

S1 Table Frequency of participants who consumed substances and/or took part in activities that may influence hormone analysis of salivary samples. (DOCX)

S1 File LC-MS/MS method. (DOCX)
