# Supplementary material for: No relationship between autistic traits and salivary testosterone concentrations in men from the general population
Source: PLoS One. 2018 Jun 14;13(6):e0198779. doi: 10.1371/journal.pone.0198779 (PMC6002020; doi:10.1371/journal.pone.0198779)
Supplement: S1 Table — (DOCX) [file pone.0198779.s001.docx]

**S1 Table. Frequency of Participants Who Consumed Substances and/or Took Part in Activities that May Influence Hormone Analysis of Salivary Samples.**

|  | Low-AQ (*n* = 22) | | Mid-AQ (*n* = 23) | | High-AQ (*n* = 22) | |
| --- | --- | --- | --- | --- | --- | --- |
| Item | Yes | No | Yes | No | Yes | No |
| Alcohol | 3 | 19 | 0 | 23 | 1 | 21 |
| Caffeine | 8 | 14 | 8 | 15 | 8 | 14 |
| Nicotine | 1 | 21 | 2 | 21 | 2 | 20 |
| Medications | 5 | 17 | 3 | 20 | 3 | 19 |
| Vigorous Activity | 4 | 18 | 4 | 19 | 3 | 19 |

*Note.* Chi-square analyses showed no significant differences in the frequency of endorsement for each factor across the three AQ groups (all *p*s > .05).
