# Supplementary material for: No relationship between autistic traits and salivary testosterone concentrations in men from the general population
Source: PLoS One. 2018 Jun 14;13(6):e0198779. doi: 10.1371/journal.pone.0198779 (PMC6002020; doi:10.1371/journal.pone.0198779)
Supplement: S1 File — (DOCX) [file pone.0198779.s002.docx]

**LC-MS/MS Method**

**Sample preparation**

Saliva samples were thawed and prepared at room temperature. First, a mixture of 250uL of saliva, 50uL of deuterated Testosterone-d5 (1ng/mL) and 1mL of ethyl acetate was vortexed for 120s and centrifuged for 10min at 3,000rpm. An upper layer of 900uL of saliva was isolated. The residue was then dried at 40°c and reconstituted in 70uL of 70% methanol. Finally, 20uL of the final mixture was injected into the liquid chromatography tandem mass spectrometry (LC-MS/MS).

**Steroid analysis**

A similar method was previously used for measuring Vitamin D from saliva samples (Clarke, Tuckey, Gorman, Holt, & Hart, 2013). The changes made to this method for testosterone analysis are outlined below.

Chromatographic separation was performed on an Agilent Technologies 2 🞨 1290 UPLC series Liquid Chromatography (LC) pumps, coupled to a 6460 Triple Quadrupole MS system operating in positive ion multiple reaction monitoring (MRM) mode. For this LC system, a 2-dimensional (2D) mode was operated, and therefore two columns were used. The first LC column was Agilent Poroshell 120 EC-C18 (2.1 🞨 50mm 🞨 2.7µm); the second column was Phenomenex C18 (150 🞨 3.0mm 🞨 2.6µm). Solvent A1: Optima Liquid Chromatography Mass Spectrometry (LCMS) water + 0.1% formic acid + 10mM Ammonium Formate; Solvent B1: Burdick and Jackson LCMS grade Methanol + 0.1% formic acid. A gradient was applied to the columns as follows: t = 0, 70% B; t = 5, 80% B; t = 7, 98% B; t = 8, 98% B; t = 8.5, 60% B; t = 9, 70% B, t = 11, 70% B. The flow rate was 0.2mL/min and the column compartment was set to 30°C. The injection volume was 20uL. Testosterone was monitored using the transitions 289.2 > 97.2 as a quantifier and 289.2 > 109.2 as a qualifier ion. Labelled Testosterone-d5 was used as an internal standard and was monitored at 294.2 > 113.2.

**Assay Precision and Sensitivity**

**Assay precision**

During the development of this methodology, 20 samples were assayed in duplicate and all coefficients of variation (CV%) < 3. Quality controls (QC) were conducted using 1:100 dilution of commercially available serum controls for testosterone (Biorad) in synthetic saliva (LGC). These were assayed at the beginning and the end of each run, and typically gave CV% < 5. Targets were derived from the kit insert and were developed using the Diasorin Radioimmunoassay. The LCMS assay was within the expected range for all 3 QC levels: 1) QC1 target 39 pM (LCMS method mean value 51.9, CV% = 16.8, n = 25), 2) QC2 target 174 pM (LCMS method mean value 201.5, CV% = 7.1, n = 25), and 3) QC3 target 319 pM (LCMS method mean value 347.3, CV% = 7.3, n = 25).

**Assay Sensitivity**

The method gave consistent signal noise ratios of > 10 at concentrations of 50 pM and this value was used as the limit of quantitation. The limit of detection for the method was 20 pM showing a consistent signal noise ratio of ≥ 3.
